# Adaptive and multi-scale feature fusion for Chinese news headline classification

**DOI:** 10.1371/journal.pone.0345779

**Published:** 2026-05-27

**Authors:** Yumin Yan

**Affiliations:** College of Literature and Journalism, Xiangtan University, Chongwen Rd, Yuhu District, Xiangtan, Hunan, China; Northeastern University, UNITED STATES OF AMERICA

## Abstract

The rapid growth of online news has led to an explosion of short Chinese headlines, which often suffer from sparse features, limited context, and high ambiguity—posing significant challenges for accurate classification. To address these issues, this paper proposes two tailored deep learning models: ERNIE-AAFF-SECNN for large-scale datasets, which enhances semantic representation via adaptive fusion of multi-layer ERNIE features and improves local feature extraction with SE-empowered CNN; and ERNIE-MSSE-DSCNN for small-scale datasets, which integrates multi-scale SE attention, depthwise separable convolutions, and adversarial training to boost robustness under data scarcity. A large number of experiments have shown that both of these models have achieved the most advanced performance. It is worth noting that the accuracy of ERNIE-AAFF-SECNN on the THUCNews and Toutiao datasets is 1.28% and 0.55% higher, respectively, than that of the lightweight SOTA model TinyBERT. The accuracy of ERNIE-MSSE-DSCNN on a 10% training dataset is 2.62% and 3.76% higher than that of the lightweight SOTA model TinyBERT, respectively. It demonstrates outstanding effectiveness under both standard and low-resource Settings. These results demonstrate that targeted architectural enhancements—such as adaptive feature fusion and multi-scale attention with adversarial training—can significantly improve the accuracy and robustness of short-text classification in practical Chinese news applications.

## 1 Introduction

With the rapid advancement of internet technology, the proliferation of major social platforms, and the extensive deployment of digital infrastructure, the speed of information generation has increased exponentially. According to the 53rd Statistical Report on Internet Development in China released by the China Internet Network Information Center (CNNIC) [1], as of December 2023, China’s internet penetration rate reached 77.5%, with 782.7 million users accessing online information via search engines, accounting for 75.7% of the total internet population. At the same time, the rapid development of emerging technologies such as big data, cloud computing, and artificial intelligence, coupled with the integration of network and media ecosystems, has led to an unprecedented surge in Chinese short texts—such as microblog posts, news headlines, and search snippets—flooding the public digital space.

Under emerging paradigms such as low-code development, collaborative governance, and AI-driven efficiency enhancement, short text classification plays a critical role in optimizing information utilization. It helps conserve computational resources, facilitates efficient information retrieval, and alleviates issues related to information overload. Compared to traditional media, online new media is characterized by high volume, strong timeliness, and rich interactivity. Effective headline classification not only supports domain-specific segmentation of full-text articles but also reduces computational overhead due to the brevity of the input text. Accurate and efficient news classification enables better content organization, improves user experience, increases readership, and fosters user engagement.

Furthermore, in-depth analysis of such textual data allows content creators and policymakers to identify emerging social concerns and public sentiment trends promptly. Starting from the fundamental task of classifying very short headlines, a variety of downstream natural language processing (NLP) applications have been derived, including recommendation systems [2], sentiment analysis [3], and automated question answering [4]. In real-world scenarios, this capability supports critical domains such as targeted advertising [5] and public opinion monitoring [6].

However, the explosive growth of news data has also introduced challenges such as difficulties in data management and reduced processing efficiency. Traditional text classification methods [7], which often rely on handcrafted rules or shallow machine learning models, not only require labor-intensive feature engineering but also struggle to handle complex semantic representations. In contrast, deep learning techniques have demonstrated significant advantages in the era of big data, enabling the automatic extraction of features from large-scale text corpora and delivering superior accuracy, generalization, and robustness [8]. In recent years, the rise of large language models (LLMs) [9] has further advanced the state of the art in headline classification.

That said, news headline classification remains a challenging task, primarily due to the extreme brevity of headlines, which are often highly condensed summaries of full articles with limited contextual cues. For instance, statistics from the NLPCC 2017 shared task corpus show that 95% of Chinese news headlines contain no more than 30 characters. This sparsity often leads to issues such as data sparsity, word polysemy, and semantic ambiguity. Existing feature learning methods for Chinese short texts often fail to adequately capture contextual meaning or compensate for limited lexical information, which in turn constrains classification performance.

## 2 Related work

Text feature extraction comprises two main components: feature processing, which prepares data for computational tasks, and feature representation, which converts unstructured text into a machine-readable format. In textual data processing, many researchers utilize external knowledge bases for feature processing [10] or focus on improving feature representation in natural language processing tasks. Feature processing involves operations such as feature selection and data augmentation, aiming to enhance the descriptive capacity of the data for prediction tasks and reduce the computational complexity of classification algorithms. In high-dimensional scenarios, effective feature processing can provide simpler and more learnable input structures, significantly improving data mining performance and supporting the practical deployment of deep learning models [11]. Although not always mandatory, techniques like data augmentation [12] and feature selection [13] are particularly valuable in large-scale applications, as they improve data quality and help models identify the most informative features. Feature selection reduces memory and CPU consumption by eliminating irrelevant, redundant, or inconsistent attributes. However, Chinese text poses unique challenges due to its lack of explicit word boundaries and unstructured nature. In short texts with limited vocabulary, this often leads to noisy training outcomes. Traditional methods emphasize keyword extraction accuracy based on word segmentation and frequency statistics, treating all words equally without considering the varying importance of different parts of speech or word-level correlations. To address this, Liu et al. [14] proposed a feature selection method that integrates part-of-speech tagging with external large-scale corpora to enrich word semantics and enhance feature representation in short texts. By selecting informative words based on their part-of-speech and mapping them to semantic concepts in the HowNet lexicon, the method improves feature coverage. However, it treats words as independent units, ignoring contextual dependencies, which may lead to semantic loss. Moreover, its reliance on large external resources increases computational costs and reduces focus on the intrinsic properties of the text.

Alternative approaches explore semantic grouping. Mendez et al. [15] grouped words into semantic topics and constructed topic-level feature vectors for machine learning tasks such as spam filtering. Zhu et al. [16] introduced a supervised part-of-speech-based feature selection method that leverages both word and news headline category information to improve classification accuracy. Data augmentation enhances model generalization by increasing data diversity and balancing distribution patterns, thereby reducing dependence on large annotated datasets and high-performance computing. While particularly beneficial for small datasets, excessive augmentation may introduce noise or increase computational load. Common strategies include synonym replacement, random insertion, word swapping, and random deletion—operations shown by Wei et al. [17] through ablation studies to effectively improve classification accuracy. The emergence of large language models has transformed data augmentation practices. Since OpenAI released ChatGPT in November 2022, users can generate high-quality synthetic data through simple instructions. Woźniak et al. [18] demonstrated that using ChatGPT for data augmentation significantly improves the performance of smaller models, enabling efficient and cost-effective development of sentiment analysis systems. From a historical perspective, early text processing relied heavily on rule-based methods. Luhn et al. [19] pioneered automated indexing by encoding text into computer-stored lexicons, improving retrieval efficiency and marking a major breakthrough in information retrieval. However, machine learning methods prior to the 2000s remained at a shallow learning level and faced challenges such as poor semantic expressiveness, high-dimensional sparse representations, loss of word order information, and data sparsity, all of which limited model performance. These traditional methods also struggled to capture contextual and structural information in text, hindering the learning of deep semantic features. Since 2010, deep learning has become the dominant approach in text classification, particularly under imbalanced training conditions. Neural network models such as Recurrent Neural Networks (RNN) [20], Convolutional Neural Networks (CNN) [21], Long Short-Term Memory networks (LSTM) [22], and FastText [23] have been widely adopted. For example, Guan Pengfei et al. [24] used a bidirectional LSTM to capture semantic information and performed parallel fusion for sentence-level sentiment analysis. Chen et al. [25] constructed a multimodal network based on an RNN architecture for document summarization and classification. Compared to traditional machine learning, deep learning offers significant advantages in automatic feature learning. It overcomes limitations such as feature sparsity, high dimensionality, and limited expressiveness, eliminates the need for manual feature engineering, and enables end-to-end learning from raw input to final output. Supported by advances in hardware acceleration, deep learning provides a powerful framework for modern natural language processing applications. To facilitate direct comparison, we summarize the main characteristics of representative approaches in Table 1.

**Table 1:** Summary of representative works.

Reference	Methodology	Limitations
Kim et al. [26]	TEXT-CNN	It restricts the visual aspect of local feature extraction, weakens the capture of global features, and fails to effectively capture the temporal information and word order relationship in the title
Zhang et al. [27]	TF-IDF + TEXT-CNN	Insufficient semantic features and implicit context relationships cannot be fully captured, resulting in inaccurate classification of short texts with similar word vectors but different contexts and less text information
Man et al. [28]	BERT + TEXT-CNN	Small datasets cannot provide sufficient information to support the training of complex models. Complex models will overfit the details in the training data, resulting in a decline in the generalization ability of the model

By analyzing and improving the shortcomings of the above literature, our contributions are as follows:

(1) We proposed a data processing strategy that adopts different processing thinking for datasets of different scales. This strategy can handle datasets of different sizes, addressing redundancy in large-scale data and overfitting issues in small-scale multi-category text classification tasks.
(2) We identified the limitations of traditional feature integration in language models and proposed an adaptive fusion mechanism that can dynamically capture the importance of multi-layer representations, thereby achieving more fine-grained and context-sensitive semantic modeling.
(3) During the model learning process of the small news headline dataset, an adversarial training strategy is adopted to enhance robustness and generalization.
(4) An improved deep classification architecture combining enhanced attention mechanisms and efficient convolutions better to capture salient features and complex dependencies in short texts.

## 3 Method

### 3.1 Current difficulties in news headline classification

(1) Difficulty in data processing: Chinese words are usually composed of multiple characters, with diversity and combinability, and improper word segmentation can cause headline ambiguity. Mainstream word segmentation preprocessing mainly relies on the generation of existing lexicons. For news headlines in specific domains, such as the “entertainment” domain, headlines containing various names and other new words, “e-sports” game names like “Stimulating Battlefield” are fixed four-character nouns, but after word segmentation, they become two different semantic nouns, especially the word “battlefield” has a strong category bias in “military” domain headlines. When the underlying lexicon is inaccurate or incomplete, semantic breaks occur. Headlines need to be unified to a predefined fixed length when input into the model, but news headlines vary greatly in length. For large datasets, too long leads to information redundancy and increased computational overhead; too short may lead to some key information being truncated, and losing semantic information.
(2) Limitations of word vectors: When processing extremely brief texts with limited information, the available contextual cues are often insufficient. In such cases, static word vectors fed into the model may fail to adequately capture semantic features and implicit contextual relationships, resulting in misclassification of short texts that have similar word vectors but differ in meaning due to sparse context. The introduction of dynamic word vectors based on the Transformer architecture has largely addressed this gap. By stacking multiple layers of self-attention and feedforward networks, the model is capable of capturing rich hierarchical structures and semantic information from textual data. Typically, word vector outputs rely solely on the final layer of the pre-trained model, or are obtained by simply averaging or concatenating all layer outputs to form text representations. However, this approach raises a critical issue: since different layers capture distinctly different types of information, such representations often fail to adequately emphasize the overall semantics of the text. As a result, the rich semantic information encoded across various layers of the model is not fully utilized.
(3) Short text classification model: TextCNN is currently a mainstream classifier for text analysis. It employs convolutional neural networks with sliding windows of varying kernel sizes to capture local text features, where each channel outputs distinct feature information extracted by different kernels. However, this architecture does not share parameters across kernels, and the fixed size of the convolution kernels limits the receptive field for local feature extraction, thereby weakening the model’s ability to capture global features. As a result, TextCNN struggles to efficiently model temporal information and word-order relationships in headlines, which can lead to an inaccurate understanding of the overall meaning. Increasing the width of the convolution kernels could, in theory, expand the receptive field, but it would also cause a sharp rise in the number of parameters—especially in large datasets—and add computational complexity that degrades performance. Furthermore, the final pooling step aggregates all channel information from the input features uniformly, which risks smoothing out certain key words and diminishing their impact. Additional limitations, such as the sparse connectivity of convolution operations, spatial invariance, and channel separation, also constrain the overall performance of the model.
(4) Model limitations: For large datasets, which can provide abundant features and patterns, deep networks demonstrate excellent performance by abstracting and combining input data features layer by layer. However, small datasets cannot supply sufficient information to support the training of such complex models. In these cases, complex models tend to overfit the nuances of the training data, resulting in reduced generalization capability. Moreover, deep networks require substantial computational resources for training and optimization, and small datasets may not fully utilize these resources, leading to inefficiency. On the other hand, when using simpler network architectures, the model may fail to adequately learn the underlying patterns and features of the data. This can cause underfitting and reduce the transparency and interpretability of the decision-making process, making it harder to understand how predictions are derived. While traditional CNN models perform strongly with large-scale datasets, they are prone to overfitting on small-scale datasets, which limits their applicability in data-scarce scenarios.
(5) Scale limitations: News headlines are typically concise, with limited vocabulary and informational content. This makes it challenging for classification models to adequately capture effective semantic information and key features. The issue is further exacerbated when working with small datasets, which constrain both the quantity and quality of available information and intensify problems of data sparsity. As a result, the features that can be learned during training are often insufficient, causing downstream neural network classifiers to fail in capturing the overall semantic meaning of headlines. Instead, these models tend to fit to noise rather than generalizable patterns, ultimately leading to overfitting.

### 3.2 Overall model structure

**Fig 1:**
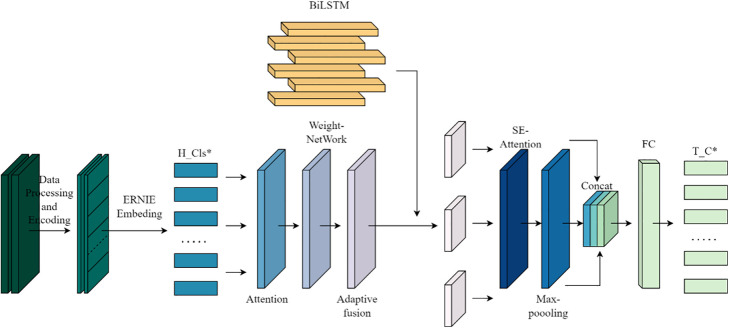
ERNIE-AAFF-SECNN network model structure.

First of all, in this experiment, the ERNIE 2.0 base model released by Baidu is used. ERNIE 2.0 is a knowledge-enhanced pre-trained language model. It adopts a continuous pre-training framework and better captures Chinese language features by introducing multi-task learning objectives at the lexical level, syntactic level and semantic level. Then,To address the difficulties (1), (2), and (3) in the previous section, we propose an extremely short Chinese news headline classification method based on the ERNIE-AAFF-SECNN network model structure. The model mainly includes three parts: data processing and encoding module, adaptive feature fusion module, and BiLSTM-SE-TextCNN classification module, as shown in Fig 1.

#### 3.2.1 *Data processing and encoding module.*

The data processing and encoding module primarily consists of two parts: data processing and ERNIE character embedding.

(1) Data Processing

For general short text classification, word-level embeddings are commonly adopted as model inputs, as they can capture meaningful lexical and syntactic information inherent in Chinese language structures. However, this approach is less effective for highly condensed texts such as news headlines, which often consist of fragmented phrases lacking full grammatical structure. Headlines typically omit function words—such as modifiers, conjunctions, and auxiliary terms—that contribute to contextual flow in standard sentences, making them structurally distinct from conventional Chinese text. Moreover, modern neural language models generate contextualized feature representations through deep architectures, where preprocessing steps like word segmentation may inadvertently break meaningful character combinations and disrupt subtle semantic patterns. In such cases, character-level modeling offers a more robust alternative. By treating individual characters as basic units, the model can directly learn how characters combine to form meaningful expressions, effectively capturing morphological and compositional regularities in headlines without relying on external segmentation tools. Character-level input also provides practical advantages in terms of model efficiency. Unlike word embeddings, which require a large embedding matrix proportional to the vocabulary size—potentially leading to high parameter counts and increased computational cost—character embeddings operate on a much smaller vocabulary (typically a few thousand Chinese characters). This significantly reduces model complexity, lowers memory usage, and accelerates training and inference. As a result, character-based representations not only preserve fine-grained semantic integrity in short, noisy headlines but also enhance computational efficiency, making them particularly suitable for real-world headline classification tasks.

Given the title sequence L, we first obtain the character length sequence W={w1,w2,...,wp}, where wi represents the i-th character of the title. Here, p denotes the maximum input length of the title. Titles shorter than p are padded with zeros, while titles exceeding p are filtered. Due to the extremely short nature of titles, to preserve their original semantics, only unrecognizable table symbols and special characters are removed to ensure the title length remains at p. Considering the varying character lengths of titles (ranging from a minimum of 7 characters to a maximum of 36 characters, with most titles around 20 characters), to avoid introducing excessive zero feature vectors during the feature extraction and classification stages, the median value between the longest title and the average title length is selected as the maximum character value p in the title sequence.

(2) ERNIE character embedding

**Fig 2:**
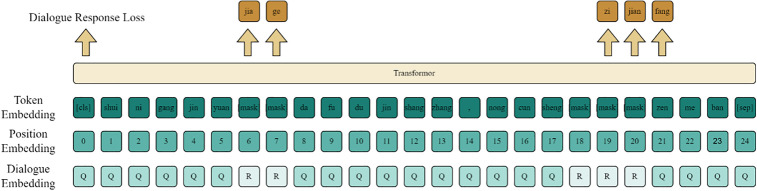
ERNIE word embedding structure diagram.

The ERNIE pre-trained model is adopted as the backbone of this framework to endow it with a superior capacity for understanding semantic and entity relationships, as well as domain-specific concepts found in headlines, such as “e-sports,” “stocks,” and “military.” This capability is further augmented by integrating entity information from knowledge graphs. The technical procedure, illustrated in Fig 2, begins with the tokenization of the headline character sequence and its subsequent mapping into ERNIE. Following a masking operation, the sequence is encoded by a Transformer to produce dynamic character vector representations.

First, the vocabulary of the ERNIE model is queried to tokenize each character of the input headline. During this process, [CLS] and [SEP] tokens are added to the beginning and end of the processed text, respectively. The [CLS] token is placed at the start of each sentence, representing the first vector of the sentence, and is primarily used for subsequent headline classification tasks. The [SEP] token serves as a separator between headlines, marking the boundaries of different headlines.

The construction of ERNIE’s character embedding vector consists of three parts: Token Embedding, Position Embedding, and Sentence Embedding. Token Embedding: Maps each character in the input headline, after being adjusted to a fixed length, into its corresponding character vector representation. Position Embedding: Uses absolute position encoding to add a fixed position vector to each character in the headline. Sentence Embedding: Converts the semantic information of the entire headline into a corresponding vector representation. This considers both global and local information of the headline to better model and understand the conversational context.

The three embeddings are combined through addition to form the character vector representation of the headline, enabling ERNIE to better understand the semantic and contextual information of the input headline and providing a comprehensive input representation for downstream tasks. The input headline sequence features are transformed into character vector sequence features *W*_*i*_={*w*_1*i*_,*w*_2*i*_,...,*w*_*ni*_} Finally, all *W*_*i*_ are concatenated to obtain the character vector matrix *W*=(*w*_1_,*w*_2_,...,*w*_*n*_).

#### 3.2.2 *Adaptive feature fusion module.*

**Fig 3:**
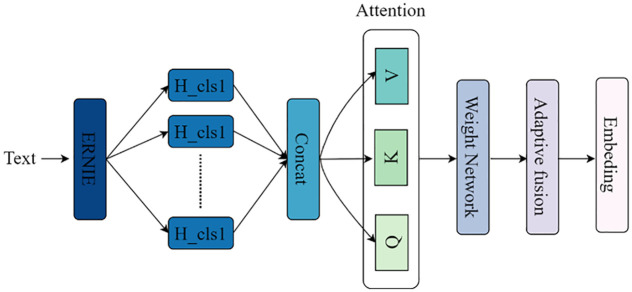
AAFF structure diagram.

The Attention-based Adaptive Feature Fusion (AAFF) module is motivated by the observation that outputs from different Transformer layers carry varying degrees of task-relevant semantic information. Instead of treating all layers equally or relying on a single aggregated representation, AAFF enables the model to dynamically learn the relative importance of each layer’s output according to the specific classification task. In our implementation, AAFF operates on the sequence of character-level feature representations generated across all layers of the ERNIE model. These multi-level outputs collectively encode contextualized semantic information spanning the entire headline, from shallow to deep linguistic patterns. The module employs a learnable attention mechanism to compute adaptive weights for each layer’s hidden states, allowing the model to emphasize the most informative levels and suppress less relevant ones. By fusing hierarchical features in a weighted manner, AAFF produces a refined, context-aware representation that preserves fine-grained semantic distinctions. This approach avoids the information loss associated with flattening or concatenating fixed-layer outputs, and instead supports flexible, data-driven integration of deep contextualized features. The architecture of AAFF is depicted in Fig 3.

(1) Input layer

ERNIE is a knowledge-enhanced character embedding representation pre-trained model, primarily employing a multi-layer self-attention bidirectional modeling structure. It utilizes residual connections for training character vectors of headline texts. The character embedding training is conducted in a progressive, layer-by-layer manner, where deeper layers produce feature outputs that more effectively represent the character feature vectors across different headlines.

**Fig 4:**
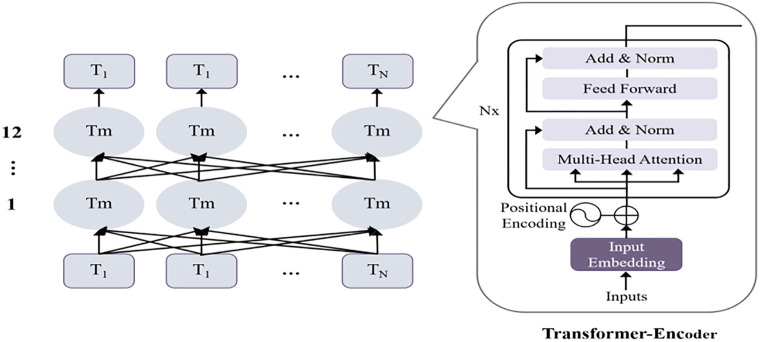
ERNIE model structure.

Thus, the last *L* layers of outputs {*H*_1_,*H*_2_,…,*H*_*t*_} from the 12-layer Transformer title character features are selected as the input layer for the AAFF module. Here, each layer’s output *H*_*i*_ ∈ *R*^*N*×*d*^ represents the series of feature representations of the *i*-th layer, where *N* is the sequence length and *d* is the feature dimension. Furthermore, the hidden features of each layer in the sequence are extracted to obtain a unified abstract representation of the hidden states for each layer. The internal structure of the ERNIE model is shown in Fig 4.

Since the first element of the ERNIE output corresponds to the classification token [CLS], which represents the semantic information of the entire headline text and contains the global feature representation of each layer’s hidden state, the hidden state sequence of [CLS] from each layer is selected as the input to the attention mechanism. This is done to extract the sequence of hidden features of each layer, considering that the same character in different headlines may contribute differently to the classification effect depending on its position. By focusing more on the overall semantics of the headline, the global abstract representation of each layer’s hidden state {*HG*_1_,*HG*_2_,...,*HG*_*k*_} is obtained, where each layer’s output *HG*_*i*_ ∈ *R*^*l*×*d*^.

Finally, the hidden states of each layer for the input headline are stacked to form a hidden state matrix *H* that includes the feature representations of each layer. This matrix serves as the core input for the AAFF module, where *H* ∈ *R*^*L*×*d*^.

(2) Attention layer

The Attention layer primarily consists of three transformation layers: Query (Q), Key (K), and Value (V) within the attention mechanism. The core idea of this module is to compute the correlations between features of different layers in each headline using the attention mechanism and dynamically adjust the weights of each layer’s features in the final fused representation based on these correlations.

First, the Q transformation layer converts the hidden state matrix, which contains the semantic feature representations of each layer of the input headline, into query vectors. These query vectors are used in the subsequent attention mechanism to match with the keys. Similarly, the K transformation layer converts the hidden state matrix output into keys, which are matched with the queries in the attention mechanism. The V transformation layer converts the hidden state matrix output into values, which are weighted according to the attention weights to obtain the fused features. The calculation formulas are shown in Equations (1)-(3).


$$\begin{array}{c} Q=W_{Q}H \end{array}$$ (1)

$$\begin{array}{c} K=W_{K}H \end{array}$$ (2)

$$\begin{array}{c} V=W_{V}H \end{array}$$ (3)

Here, *W*_*Q*_, *W*_*K*_, and *W*_*V*_ ∈ *R*^*d*×*d*^ are learnable parameter matrices that map features into a new space for participation in the attention computation.

The attention mechanism is used to evaluate the interrelationships and relative importance between the outputs of the hidden state matrix, as well as the interrelationships and relative importance between the hidden states of each layer of the input text. The final attention weights are obtained by normalizing with the Softmax function, as shown in Equations (4)-(5).


$$\begin{array}{c} A_{ij}=\frac{Q_{i}\overset{T}{\underset{j}{K}}}{\sqrt{d}}\; \end{array}$$ (4)

$$\begin{array}{c} \alpha_{ij}=Softmax\left(A_{ij}\right) \end{array}$$ (5)

Here, *A*_*ij*_ ∈ *R*^*N*×*N*^ represents the attention scores between the *i*-th and *j*-th layers, and *d* is the dimension of the query *Q* and key *K*.

The hidden state vector *HA*_*i*_ for each layer, after attention representation, is calculated using the attention weights and the weighted sum of the values, as shown in Equation (6).


$$\begin{array}{c} HA_{i}=\sum_{j=1}^{L}\alpha_{ij}V_{J} \end{array}$$ (6)

(3) Weight network and adaptive fusion

At the end of the module, a Weight network is introduced to further refine the control over the contribution of each layer, allowing the model to learn a more complex and sophisticated strategy for information fusion across layers. The weighted network is implemented using two fully connected layers with ReLU activation and a Softmax layer, with the output layer dimension being *L*, corresponding to the *L* layers of the ERNIE model. Its goal is to automatically learn the contribution of each layer’s character vectors to the final features based on the hidden state vectors *HA*_*i*_ computed by the Attention layer, dynamically adjusting the importance of each layer’s Transformer features during the fusion process.

The weighted hidden state vectors *HA*_*i*_ from all layers, computed by the Attention layer, are used as input to the weight learning network to further adjust the weight of each layer’s feature output in the final fused character representation. The final fused features can be represented as the weighted sum of all layer outputs.

First, the weighted vectors *HA*_*i*_ from all layers are concatenated and input into the weight learning network, as shown in Equation (7):


$$\begin{array}{c} w=Softmax\left(W\cdot HA_{i}\right) \end{array}$$ (7)

Where *W* represents the learnable parameters of the fully connected layer.

Finally, the weighted sum of all layer outputs *HA*_*i*_ is computed to obtain the final fused features *H*_*fused*_. The network’s output *w* ∈ *R*^*L*^ is passed through the Softmax layer to obtain the final weight *w*_*i*_ for each layer, used for the weighted summation of the character vector features, as shown in Equation (8):


$$\begin{array}{c} H_{\text{fused}}=\sum_{i=1}^{l}w_{i}\cdot HA_{\text{i}} \end{array}$$ (8)

#### 3.2.3 BiLSTM-SE-CNN classification module.

This module takes the output of ERNIE character embeddings, which have undergone adaptive feature fusion, as its input and constructs the BiLSTM-SE-CNN classification module.

(1) BiLSTM layer

**Fig 5:**
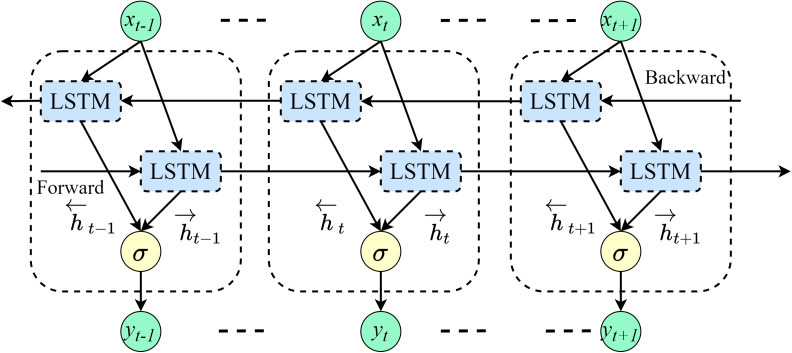
BiLSTM network model structure.

The internal structure of LSTM includes an input gate, a forget gate, an output gate, and a cell state. The cell state determines the information to be retained and the historical text information, while the gate structures precisely control the cell state based on specific signals, regulating information by selectively deleting or adding information. BiLSTM combines forward LSTM and backward LSTM, addressing issues such as vanishing or exploding gradients encountered in news text classification tasks. The network structure is illustrated in Fig 5.

In the ERNIE-AAFF-SECNN model, a two-layer BiLSTM is employed to capture more complex contextual information within the headlines. This hierarchical approach allows the model to learn higher-level features, which are used to analyze the sentence structure and contextual information in news headlines. The BiLSTM serves as a bridge between the character embeddings and the downstream convolutional network.

For the input news headline sequence *H*_*fused*_=[*X*_1_, *X*_2_,  ..., *X*_n_], the LSTM unit’s computation at each time step *t* consists of four gates. By recording the relationships of the information, a value between 0 and 1 is output to the cell state *C*_*t*-*l*_ using the Sigmoid function, as shown in Equation (9):


$$\begin{array}{c} f_{t}=\sigma \left(W_{f}\cdot \left[h_{t-1},X_{t}\right]+b_{i}\right) \end{array}$$ (9)

The input gate determines the extent to which information passes through, helping the model selectively focus on important information in the current input text. The Sigmoid function processes the current input, outputting a value between 0 and 1, and the tanh layer generates a new candidate value vector *g* to be stored in the state, as shown in Equations (10)-(11):


$$\begin{array}{c} i_{t}=\sigma \left(W_{f}\cdot \left[h_{t-1},X_{t}\right]+b_{i}\right)\; \end{array}$$ (10)

$$\begin{array}{c} g=tanh\left(W_{C}\cdot \left[h_{t-1},X_{t}\right]+b_{C}\right) \end{array}$$ (11)

The memory unit updates the old memory unit *C*_*t*-1_ to the new memory unit *C*_*t*_, as shown in Equation (12):


$$\begin{array}{c} C_{t}=f_{t}*C_{t-1}+i_{t}\cdot g \end{array}$$ (12)

The output gate uses the Sigmoid layer to output the current ot, and then the tanh function processes the state to obtain the final hidden state ht, resulting in the final output value, as shown in Equations (13)-(14).


$$\begin{array}{c} o_{t}=\sigma \left(W_{O}\cdot \left[h_{t-1},h_{t}\right]+b_{o}\right) \end{array}$$ (13)

$$\begin{array}{c} h_{t}=o_{t}*tanh\left(C_{t}\right) \end{array}$$ (14)

Here, *W* is the weight matrix, *b* is the bias vector, *σ* is the Sigmoid neural network layer, and * denotes element-wise multiplication.

During the classification process, BiLSTM processes the sequence using two independent LSTMs: one processes the sequence in the normal order, and the other processes it in the reverse order. Finally, at each time step *t*, a richer representation containing contextual information from both directions of the headline is formed, as shown in Equations (15)-(17):


$$\begin{array}{c} {\vec{h}}_{t}=LSTM\left(h_{t-1}W_{t},c_{t-1}\right),t\in \left[1,T\right] \end{array}$$ (15)

$$\begin{array}{c} {\overset{\leftarrow }{h}}_{t}=LSTM\left(h_{t+1}W_{t},c_{t+1}\right),t\in \left[1,T\right] \end{array}$$ (16)

$$\begin{array}{c} Y_{t}=\left[{\vec{h}}_{t},{\overset{―}{h}}_{t}\right] \end{array}$$ (17)

(2) SE-CNN

**Fig 6:**
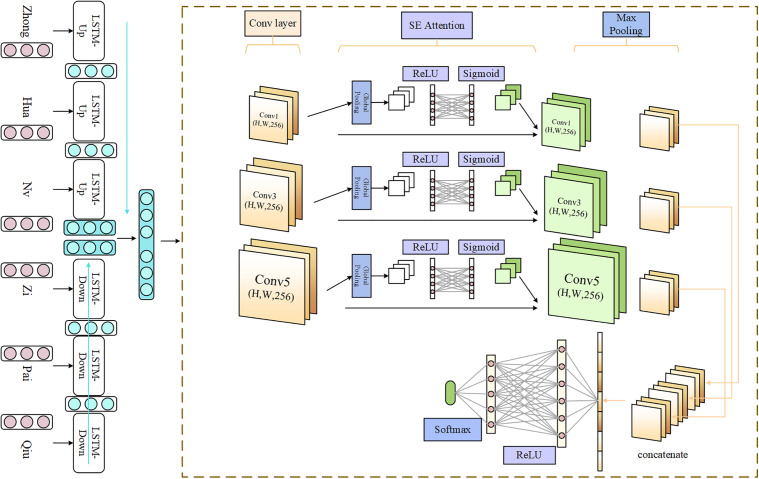
BiLSTM-SE-CNN network model architecture.

To overcome the limitations of standard convolution—such as sparse connectivity, spatial invariance, and fixed channel weighting, which hinder performance on short headline texts—we incorporate the Squeeze-and-Excitation (SE) attention mechanism into TextCNN. The enhanced model applies SE to reweight the hidden state outputs from BiLSTM, enabling adaptive emphasis on informative features. This allows the convolutional layer to focus on salient local patterns in the headline. A two-layer fully connected network with ReLU activation is then used to adjust feature dimensions and boost representational capacity for classification. The overall architecture of the BiLSTM-SE-CNN model is shown in Fig 6.

1) Convolutional layer

The convolutional layer employs three different kernel sizes (Conv1, Conv3, Conv5) to capture textual features between sequences of varying distances in the headline. The kernel sizes are 1, 3, and 5, respectively, and they operate in a sliding window manner to extract feature maps from the hidden sequence *y*_*t*_ obtained from the previous layer, as shown in Equation (18):


$$\begin{array}{c} z_{i}=f\left(W\cdot Y_{i,i+h-1}+b\right) \end{array}$$ (18)

Here, *W* and *b* are the weights and bias terms of the convolutional kernel, *Y*_*i*,*j*+*h*-1_ is the feature vector with a window size of *h*, and *f* is the ReLU activation function.

2) SE-attention layer

**Fig 7:**
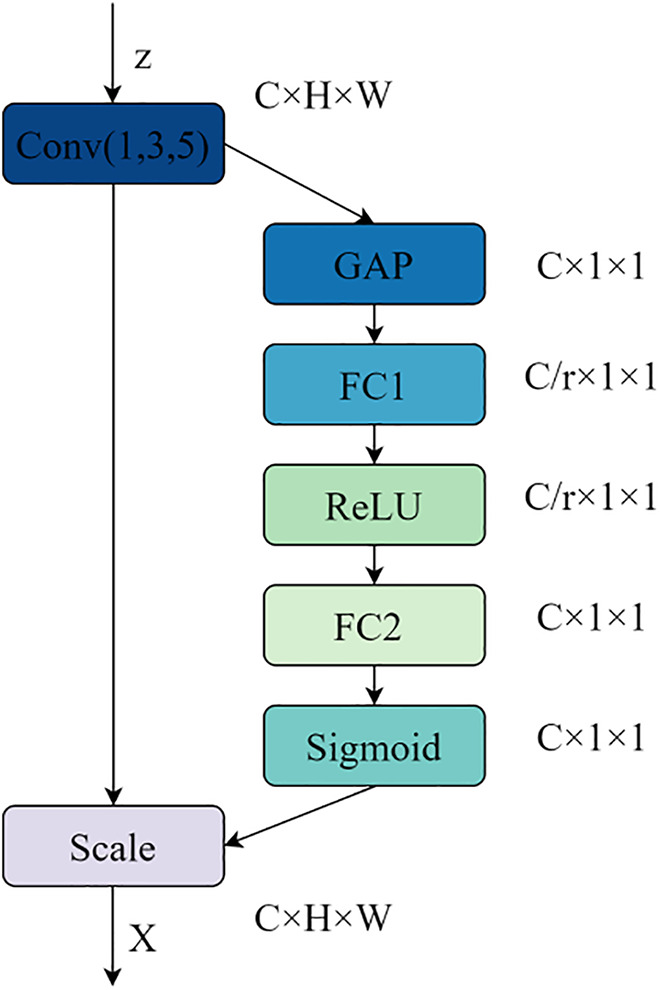
SE-Attention network model architecture.

Feature maps are extracted from the convolved *Z*_*i*_ sequence distribution. For feature maps extracted using three different kernel sizes, different weights are assigned to the feature maps of each channel, emphasizing important text in the headline while suppressing less important text. The structure of the SE-Attention mechanism is illustrated in Fig 7.

First, the *Z*_*i*_ sequence undergoes global average pooling (GAP) to produce the output feature map GAP_out_. This compresses the spatial dimensions of the feature map, achieving a global low-dimensional embedding. This is equivalent to a single value representing the global receptive field of the channel, transforming the feature map size from *C* × *H* × *W* to C × 1 × 1, as shown in Equation (19):


$$\begin{array}{c} {\text{GAP}}_{\text{out}}=\frac{\text{1}}{H\times W}\sum_{i=1}^{H}\sum_{j=1}^{W}Z_{i}(I=i,j) \end{array}$$ (19)

Next, the feature map passes through two fully connected layers, FC1 and FC2. FC1 compresses the channel dimension to reduce the number of parameters and computational load, while FC2 restores the channel dimension to its original size, ensuring consistency in the subsequent weighting dimensions, as shown in Equations (20)-(21):


$$\begin{array}{c} {\text{FCl}}_{\text{out}}=ReLU\left({\text{GAP}}_{\text{out}}\times w_{\text{1}}+b_{\text{1}}\right) \end{array}$$ (20)

$$\begin{array}{c} \text{FC}{\text{2}}_{\text{out}}=Sigmoid\left(\text{FC}{\text{1}}_{\text{out}}\times w_{\text{2}}+b_{\text{2}}\right) \end{array}$$ (21)

Finally, the output is multiplied by the input convolutional *Z*_*i*_ sequence to obtain the weighted sequence *SZ*_*i*_.

3) Pooling layer and double fully connected layer (FC)

Max pooling is employed to extract the maximum feature value within each pooling region of every channel, as shown in Equation (22).


$$\begin{array}{c} SZ_{max}=max\left(SZ_{i}\right) \end{array}$$ (22)

Among them, max represents taking the maximum value.

The vectors obtained after max pooling for each channel are concatenated to produce a high-dimensional aggregated feature vector *SZ*.

To adapt to the multi-category classification task for Chinese news headlines, a double fully connected layer (FC) is implemented, consisting of two fully connected layers (*fc*_1_, *fc*_2_) and a ReLU nonlinear activation function. First, the high-dimensional feature vector *SZ* is mapped to a relatively lower-dimensional feature vector using the first fully connected layer *fc*_1_, achieving dimension transformation and feature integration. Next, a ReLU nonlinear activation function is introduced between the two fully connected layers to enhance the model’s expressive power, allowing it to learn more complex feature mappings and better adapt to nonlinear patterns in the data. Finally, the second fully connected layer *fc*_2_ serves as the output layer, mapping the input features to different categories, with the number of neurons equal to the number of classes in the classification task.

The model passes through a Dropout layer to mitigate overfitting, and the Softmax function is used to output the probability distribution of headline categories.

### 3.3 *Overall model structure*

**Fig 8:**
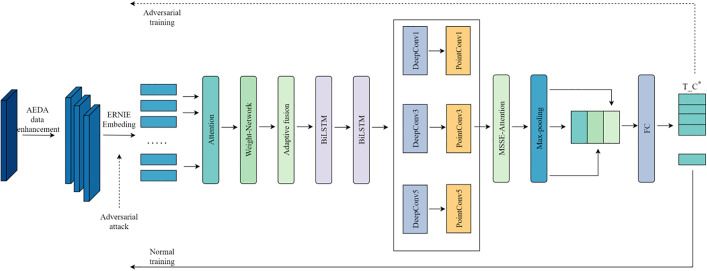
ERNIE-MSSE-DSCNN network model structure.

To address the difficulties (4) and (5) in the 3.1 section, we propose a classification method for small datasets based on the ERNIE-MSSE-DSCNN network model structure, as illustrated in Fig 8.

#### 3.3.1 *Improved AEDA word-level data augmentation.*

**Fig 9:**
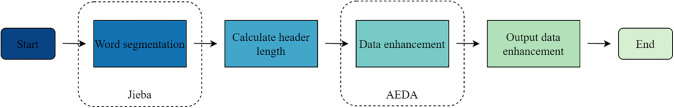
Data enhances processes.

To address the characteristics of small-scale extremely short Chinese news headline datasets, an improved AEDA (Adversarial and Easy Data Augmentation) word-level data augmentation method is proposed. The AEDA algorithm is utilized to mine the content of the headlines themselves for data augmentation. The specific workflow of the data augmentation process is illustrated in Fig 9.

First, jieba is used as the Chinese word segmentation tool to tokenize the input headlines and further segment long words to expand the headlines. The entire segmentation process is based on preserving part-of-speech (POS) tags, including nouns, gerunds, verbs, adjectives (content words), and conjunctions (function words).

After reading the POS-based segmentation results, each word is separated by a space. The AEDA algorithm is then applied to randomly insert a specified number *n* of punctuation marks at the spaces. For each headline, the length of the word sequence *l* is calculated to determine the number of punctuation marks *n* to be inserted. The positions for inserting punctuation marks are randomly assigned, consistent with the value of *n*. This ensures that the complexity of the sentence is increased without adding too many punctuation marks, which could otherwise overly disrupt the semantic information of the sentence. The formula for calculating the number of punctuation marks to be added is shown in Equation (23).


$$\begin{array}{c} n=rand\left(1,\frac{\text{1}}{\text{3}}\right) \end{array}$$ (23)

Finally, Chinese punctuation marks are inserted at random positions, with the punctuation marks randomly selected from the set {“,”,“:”,“。”,“;”}. This generates *n* augmented texts, which are then merged with the original text to provide a training set augmented by *n* times for subsequent training. A 2x augmentation is chosen, and an example of the data augmentation is shown in Table 2.

**Table 2:** Data augmentation example table.

	Title content
Original Title-1	What are some “cold knowledge” tricks in the game PUBG to deceive newbies?
Original Title-1 + AEDA	· In · · · PUBG · · · there · · · are · · · some · · · tricks · · · to · · · deceive · · · newbies · · · with · · · “cold · · · knowledge” · · ·?
Original Title-2	What to do if the engine compartment is very dirty? An old driver teaches you a trick, and the dust will be gone in 3 minutes.
Original Title-2 + AEDA	The· engine· compartment· is·· very· dirty·· what· to· do · · ·?· An· old· driver· teaches·· you·· a· trick · · · and·· the· dust·· will· be· gone·· in·· 3·· minutes.

#### 3.3.2 *MSSE-DSCNN module.*

**Fig 10:**
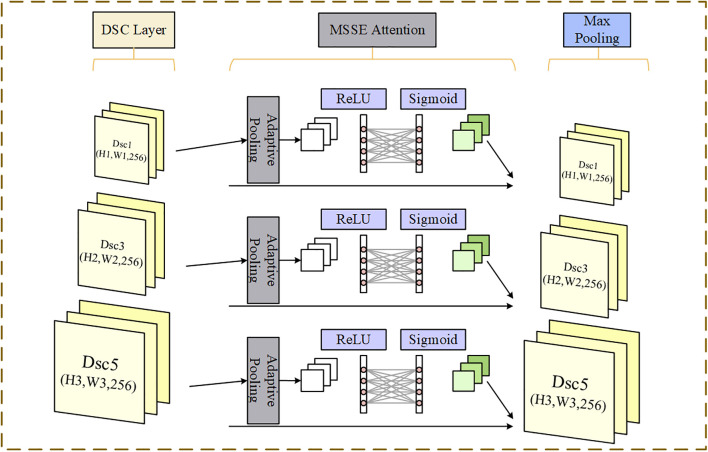
The module structure of MSSE-DSCNN.

This module utilizes the ERNIE pre-trained model with adaptive feature fusion proposed in the previous subsection to perform character embedding on Chinese headline texts, generating dynamic character vector representations. A two-layer BiLSTM network is employed to extract global features from the text. The input headline sequence features are transformed into character vector sequence features *Wi*=(*w*_1*i*_,*w*_2*i*_,...,*w*_*ni*_), and all *Wi* are concatenated into the matrix *W*=(*W*_1_,*W*_2_,...,*W*_*n*_), which represents the ERNIE character vectors. The structure of the MSSE-DSCNN module is illustrated in Fig 10.

Using 10% of the standard dataset for training, although traditional Convolutional Neural Networks (CNNs) perform well on large datasets, their numerous parameters and computational complexity often lead to overfitting when directly applied to such compact datasets for model training. This results in insufficient feature capture capability, thereby limiting their applicability to small-scale data.

**Fig 11:**
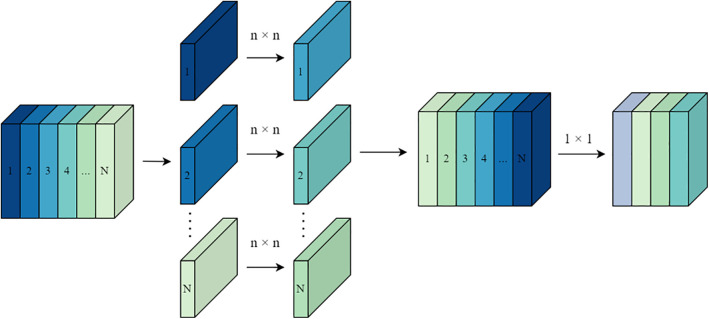
Depthwise separable convolutional structures.

Depthwise separable convolution addresses this by decomposing the traditional convolution operation into two sequential processes: depthwise convolution and pointwise convolution. In the depthwise convolution step, each input channel corresponding to the three different kernel sizes is independently convolved with a single kernel, enabling feature extraction within each channel. Subsequently, in the pointwise convolution step, a 1 × 1 convolution kernel is used to linearly combine features from different channels, generating new output feature maps. The structural model diagram is shown in Fig 11.

By adopting the depthwise separable convolution architecture and replacing the standard convolutional layers in traditional CNN models, the number of parameters and computational complexity of the model are reduced, mitigating the risk of overfitting and thereby optimizing the performance of classification tasks on small-scale Chinese headline datasets. The results indicate that this approach not only effectively reduces the computational load of the model but also enhances its ability to process small datasets, maintaining high accuracy in classification outcomes.

First, the feature maps of the headline text, after passing through the BiLSTM layer, are input into the depthwise convolution step to capture features in the spatial dimension. For each input channel, depthwise convolution independently applies a convolution kernel, producing *C* output channels for *C* input channels. Each output channel is the result of the convolution between the corresponding input channel and the convolution kernel, as expressed in Equation (24).


$$\begin{array}{c} Y_{c}=X_{c}*K_{c} \end{array}$$ (24)

Here, *Y*_*c*_ is the *c*-th channel of the output feature map, *X*_*c*_ is the *c*-th channel of the input feature map, *K*_*c*_ is the convolution kernel applied to the *c*-th channel, and * represents the convolution operation.

After depthwise convolution, pointwise convolution uses a 1 × 1 convolution kernel to linearly combine the results of the depthwise convolution and the headline text information from different channels, generating a new feature map, as shown in Equation (25):


$$\begin{array}{c} Z=Y*K^{\left(1\times 1\right)} \end{array}$$ (25)

Here, *Z* is the final output feature map, *Y* is the set of all output channels from the depthwise convolution, and *K*^(1 × 1)^ is the 1 × 1 convolution kernel.

The computational complexity of depthwise convolution is shown in Equation (26).


$$\begin{array}{c} O\left(C_{in}\times K^{\text{2}}\times H\times W\right) \end{array}$$ (26)

Here, *C*_*m*_ is the number of input channels, *H* is the height of the input feature map, *W* is the width of the input feature map, and *K* is the size of the convolution kernel.

The computational complexity of pointwise convolution is shown in Equation (27).


$$\begin{array}{c} O\left(C_{\text{in}}\times C_{\text{OUT}}\times H\times W\right) \end{array}$$ (27)

Here, *C*_*out*_ is the number of output channels.

Therefore, the total computational complexity of depthwise separable convolution can be expressed as Equation (28).


$$\begin{array}{c} C_{in}\times K^{\text{2}}\times H\times W+C_{in}\times C_{out}\times H\times W \end{array}$$ (28)

The ratio of the computational complexity of depthwise separable convolution to that of traditional convolution is shown in Equation (29).


$$\begin{array}{c} \frac{C_{\text{in}}\times K^{\text{2}}\times H\times W+C_{\text{in}}\times C_{\text{out}}\times H\times W}{C_{\text{in}}\times K^{\text{2}}\times H\times W\times C_{\text{out}}}=\frac{\text{1}}{C_{\text{out}}}+\frac{\text{1}}{K^{\text{2}}} \end{array}$$ (29)

Here, *C*_*in*_ represents the number of input channels, *C*_*out*_ denotes the number of output channels, *H* is the height of the input feature map, *W* is the width of the input feature map, and *K* is the size of the convolution kernel.

As demonstrated by Equations (27)-(29), the introduction of depthwise separable convolution can reduce the number of parameters and computational load of the model when processing small datasets, thereby improving computational efficiency. Additionally, depthwise separable convolution helps mitigate the risk of overfitting and enhances the speed of model training and inference, making the model more suitable for real-time application scenarios.

For small-scale Chinese news headline datasets, a multi-scale SE (Squeeze-and-Excitation) attention mechanism module, referred to as the MSSE module, is proposed. This module integrates global information from different scales to enhance the model’s feature representation capability. It learns the importance weights of feature channels to dynamically reweight the input feature maps, emphasizing important features while suppressing unimportant ones, thereby improving the model’s ability to capture key information and its overall performance.

**Fig 12:**
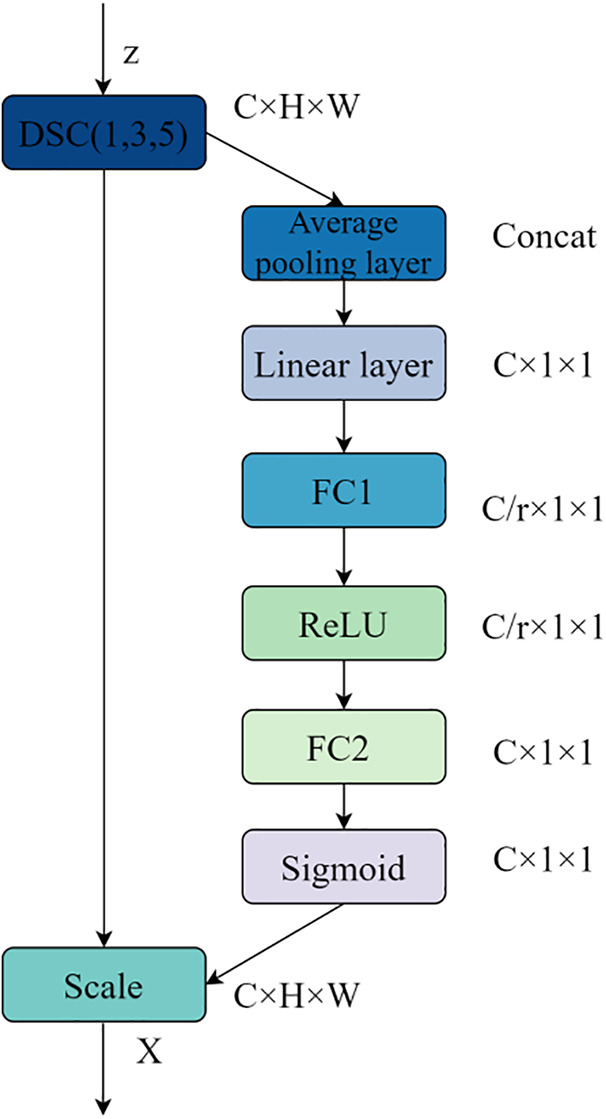
Architecture of MSSE attention mechanism.

Unlike the traditional SE attention mechanism, which relies solely on single-scale global average pooling, the MSSE module introduces a multi-scale global average pooling strategy. MSSE can capture feature information from headlines at different spatial scales, enriching and strengthening the model’s text feature representation capability and more accurately assessing the importance of each channel. This not only helps the model emphasize the expression of key features but also effectively suppresses the influence of unimportant features, further enhancing the model’s performance. By comprehensively learning and utilizing these multi-scale features, the MSSE strategy for multi-scale feature fusion endows the model with greater robustness in feature extraction and representation. The structure of the MSSE attention mechanism is illustrated in Fig 12.

First, the MSSE module applies a series of multi-scale global average pooling operations to the input feature maps processed by depthwise separable convolution with three different kernel sizes. This captures contextual information across varying ranges, as shown in Equation (30).


$$\begin{array}{c} X_{p_{s}}=P_{s}\left(X\right),s\in \left\{1,3,5,7,9\right\} \end{array}$$ (30)

Here, *s* represents different pooling kernel sizes, and $X_{p_{s}}$represents the pooled features corresponding to different pooling kernels.

The multi-scale global average pooling operation performs average pooling for each scale *s* to obtain contextual information at different scales. Subsequently, all multi-scale pooled feature maps are concatenated along the channel dimension to form a fused feature map *X*_*p*_, serving as a comprehensive feature representation, as shown in Equation (31):


$$\begin{array}{c} X_{p}=Concat\left(X_{p},X_{p_{s}},X_{p_{s}},X_{p_{t}},X_{p_{s}}\right) \end{array}$$ (31)

Next, the fused multi-scale feature map is transformed into a channel descriptor feature *z* through a linear transformation layer, as shown in Equation (32):


$$\begin{array}{c} {\text{z=X}}_{\text{p}}\text{W+b} \end{array}$$ (32)

Here, *W* ∈ *R*^*d*×*d*^ is the weight matrix of the linear transformation layer, *b* ∈ *R*^*d*^ is the bias term, *d* represents the transformed feature dimension, and *z* ∈ *R*^*n*×*d*^ is the feature representation obtained after the linear transformation.

Following this, a fully connected network with ReLU and Sigmoid activation functions is used to learn the importance weights for each channel, as shown in Equation (33):


$$\begin{array}{c} w=\sigma (FC_{\text{2}}\left(\delta \left(FC_{\text{1}}\left(z\right)\right)\right) \end{array}$$ (33)

Here, *σ* is the Sigmoid activation function, *δ* is the ReLU activation function, and FC_1_ and FC_2_ are the fully connected layers.

Finally, the learned weights are used to reweight the original input feature map, resulting in the final weighted feature map X′, which highlights the feature channels with more informative headline text, as shown in Equation (34):


$$\begin{array}{c} X^{\prime }=w\cdot X \end{array}$$ (34)

By integrating feature information from different scales, the MSSE module provides the model with a more comprehensive and enriched feature representation. It not only captures fine-grained detailed features but also captures coarse-grained contextual information. This multi-level feature capture approach helps the model fully learn from small-scale Chinese headline datasets, extracting as many useful features as possible, thereby improving the model’s generalization ability and reducing the risk of overfitting.

### 3.3 FGM strategy for adversarial training

Training deep learning models on small datasets often results in overfitting, as the limited amount of data makes it difficult for the model to learn meaningful patterns, causing it to fit noise instead. This issue is especially severe in tasks such as classifying very short Chinese news headlines, where the model needs to capture fine-grained linguistic differences and therefore requires strong generalization capability. To address this problem, we apply adversarial training using the Fast Gradient Method (FGM). FGM calculates the gradient of the loss with respect to the character embeddings in ERNIE and adds a small, gradient-aligned perturbation to these embeddings, creating adversarial samples. The model is then trained to reduce the loss on these perturbed inputs, encouraging more stable and robust learning. By exposing the model to slight variations that mimic real-world linguistic fluctuations and potential adversarial disturbances, FGM strengthens its resistance to overfitting. This approach improves generalization performance on small-scale headline classification without introducing significant computational overhead.

**Fig 13:**
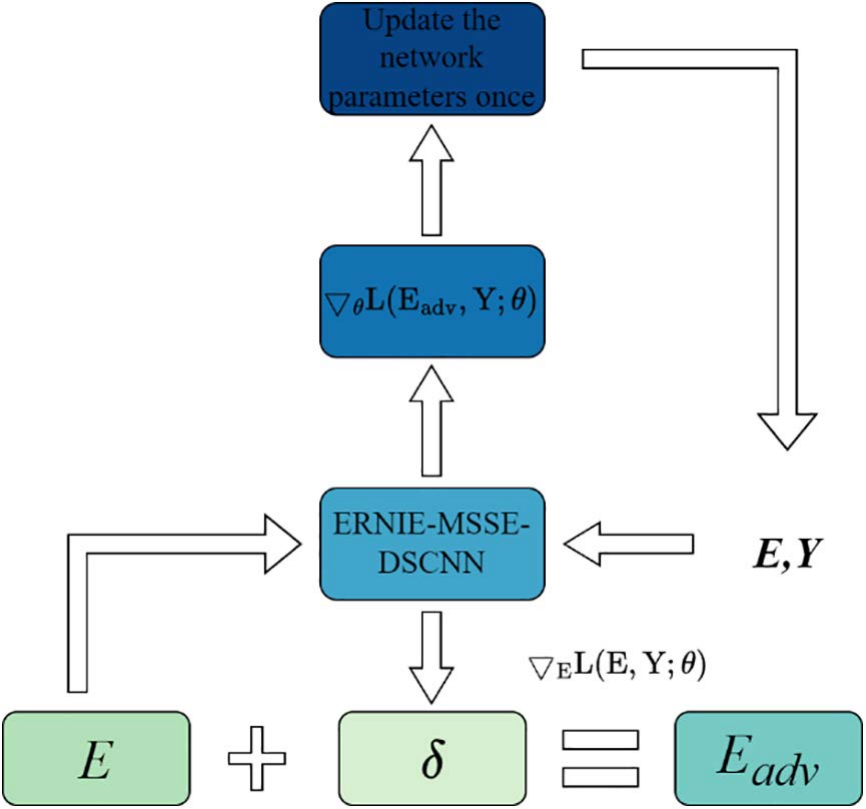
FGM adversarial training steps.

The adversarial training strategy of FGM follows the training steps illustrated in Fig 13.

First, for the given model loss *L*, compute the gradient of the loss with respect to the model’s input scalar equal to the embedding *E*. Let the model’s input word embedding be *E* ∈ *R*^*n*×*d*^, then the gradient *g* is as shown in Equation (35):


$$\begin{array}{c} g=\nabla_{E}\text{ L}(E,Y;\theta ) \end{array}$$ (35)

Here, *n* is the sequence length, *d* is the word embedding dimension, *θ* represents the model parameters, *Y* is the input label, and ∇_*E*_*L*(*E*,*Y*;*θ*) denotes the gradient of the loss *L* with respect to the word embedding *E*.

Next, based on the computed gradient *g*, generate a small perturbation that is sufficient to influence the model’s predictions. This perturbation is in the direction of the gradient’s sign, and its magnitude is controlled by the hyperparameter *k* to ensure the perturbation is minimal. The adversarial perturbation *δ* is as shown in Equation (36).


$$\begin{array}{c} \delta =\varepsilon \cdot sign\left(g\right) \end{array}$$ (36)

Here, *ε* is a predefined small constant, and sign(*g*) represents the sign function of the gradient *g*.

The adversarial perturbation *δ* is added to the original word embedding *E* to generate a new adversarial word embedding *E*_*adv*_, as shown in Equation (37):


$$\begin{array}{c} E_{adv}=E+\delta \end{array}$$ (37)

In this way, the generated adversarial samples can simulate potential worst-case scenarios based on the original headline data, helping the model learn more robust feature representations. Finally, the adversarial samples *E*_*adv*_ are used to perform forward propagation again, and the loss *L*_*adv*_ computed. The model parameters *θ* are then updated based on this loss, as shown in Equation (38):


$$\begin{array}{c} \theta =\theta -\alpha \nabla_{\theta }\text{L}\left(E_{adv},Y;\theta \right) \end{array}$$ (38)

Here, *α* is the learning rate, and ∇_*θ*_*L*(*E*_*adv*_,*Y*;*θ*) represents the gradient of the loss *L*_*adv*_ with respect to the model parameters *θ* on the adversarial samples.

Throughout the process, the model parameters are optimized using the Opt optimizer to minimize the loss *L*_*adv*_.

Finally, the word embedding vectors of the model input are restored to their pre-perturbation state, and the model after adversarial training is output.

## 4 Experimental results analysis and evaluation metrics

### 4.1 *Experimental parameters*

The experimental parameters of this paper are shown in Table 3.

**Table 3:** Parameter settings.

Parameters	Setting
Maximum sentence length	32
Hidden size	768
Dropout	0.1
Loss	Cross_entropy
Learning rate	5e-5
Optimizer	Adam

### 4.2 Datasets

To validate the classification performance of the proposed ERNIE-AAFF-SECNN model on large datasets, comparative experiments were conducted on two large datasets: the 10-category THUCNews Chinese news headline dataset and the 13-category Today’s Headlines dataset. Detailed information about the datasets and their headline content across various categories are presented in Tables 4 and 5.

**Table 4:** Example of today’s headline news corpus.

Category label	Topic	Sample headline content
3	Finance	Why is the salary level in Shandong much lower than that in several southern provinces, especially in terms of housing provident funds?
6	Education	Everyone says art school entrance exams are difficult. How can one secure 25 admission certificates with an acceptance rate of only 1.6%?
12	E-sports	The descendants of Zongbu, the deity from myths and legends in “Tales of the Nine States”
1	Entertainment	Li Yuchun seems to have fallen in love with dresses now. Every dress she wears is so stunning, truly beautiful.

**Table 5:** The THUCNews datasets and today’s headlines datasets dataset corpus.

Dataset name	Number of categories	Included categories	Training set size per category	Validation set size per category	Test set size per category	Average title length	Shortest title length	Longest title length
THUCNews Dataset	10	Finance, Real Estate, Stocks, Education, Technology, Society, Politics, Sports, Games, Entertainment	18,000	1,000	1,000	17	6	28
Toutiao Dataset	13	Culture, Entertainment, Sports, Finance, Real Estate, Cars, Education, Technology, Military, Tourism, International, Agriculture, E-sports	15000	1000	1000	20	7	36

To validate the classification performance of the ERNIE-MSSE-DSCNN model on small datasets, a new small training set was constructed by randomly sampling 10% of the 10-category THUCNews large Chinese news headline dataset and the 13-category Toutiao large Chinese news headline dataset. Comparative experiments were conducted using this small dataset, with specific details provided in Table 6.

**Table 6:** The dataset corpus of 10% of the THUCNews datasets and Today’s Headlines datasets.

Dataset name	Number of categories	Included categories	10% Dataset training set size per category	Validation set size per category	Test set size per category	Average title length	Shortest title length	Longest title length
THUCNews Dataset	10	Finance, Real Estate, Stocks, Education, Technology, Society, Politics, Sports, Games, Entertainment	1800	1000	1000	17	6	26
Toutiao Dataset	13	Culture, Entertainment, Sports, Finance, Real Estate, Cars, Education, Technology, Military, Tourism, International, Agriculture, E-sports	1500	1000	1000	20	6	28

### 4.3 Evaluation metrics

The experiments use four evaluation metrics—accuracy, precision, recall, and F1 score—to assess the classification performance of the model. The confusion matrix is presented in Table 7.

**Table 7:** Confusion matrix.

	Actually belongs to this class	Actually does not belong to this class
Predicted to Belong	TP(True Positive)	FP(False Positive)
Predicted Not to Belong	FN(False Negative)	TN(True Negative)

(1) Accuracy: Accuracy refers to the proportion of correctly predicted samples out of the total samples. The calculation formula is as shown in Equation (39):

$$\begin{array}{c} \text{Accuracy}=\frac{TP+TN}{TP+FP+FN+TN} \end{array}$$ (39)

(2) Precision: Precision refers to the proportion of samples predicted as positive that are actually positive out of all samples predicted as positive. The calculation formula is as shown in Equation (40):

$$\begin{array}{c} \text{Precision}=\frac{TP}{TP+FP} \end{array}$$ (40)

(3) Recall: Recall refers to the proportion of samples predicted as positive that are actually positive out of all actual positive samples. The calculation formula is as shown in Equation (41):

$$\begin{array}{c} \text{Recall}=\frac{TP}{TP+FN} \end{array}$$ (41)

(4) F1-Score: The F1-Score is the harmonic mean of precision and recall. The calculation formula is as shown in Equation (42):

$$\begin{array}{c} F1=\frac{2\times \text{Precision}\times \text{Recall}}{\text{Precision}+\text{Recall}} \end{array}$$ (42)

### 4.4 Analysis of experimental results on large datasets

#### 4.4.1 *Analysis of comparative experimental results.*

(1) Comparative results of classification experiments on the THUCNews dataset: On the 10-category THUCNews dataset, hybrid models with different inputs and model structures were used for ensemble training. The prediction results were evaluated using four metrics: precision (P), recall (R), F1 score (F1), and accuracy (Acc).

As shown in Table 8, the proposed ERNIE-AAFF-SECNN model achieves the highest classification performance among all evaluated models, with precision, recall, F1 score, and accuracy reaching 95.09%, 95.09%, 95.08%, and 95.09%, respectively—surpassing all baseline models. Compared to the top-performing baseline, ERNIE, the proposed model improves precision by 0.78%, recall and accuracy by 0.82%, and F1 score by 0.81%. Relative to ERNIE-CNN, which lacks the enhanced CNN architecture, gains are even more pronounced: precision increases by 0.91%, recall and accuracy by 0.94%, and F1 by 0.92%.

**Table 8:** Experimental results of different models in THUCnews.

Model	P %	R %	F1%	Acc %
TextCNN	90.93	90.86	90.87	90.86
TextRNN	90.76	90.71	90.69	90.71
DPCNN	90.58	90.50	90.47	90.50
FastText	91.82	91.79	91.80	91.79
BERT	93.87	93.87	93.86	93.87
BERT-CNN	93.89	93.85	93.83	93.85
TinyBERT	93.82	93.81	93.82	93.81
ALBERT	93.99	93.92	93.94	93.92
MobileBERT	93.85	93.82	93.83	93.83
DistilBERT	93.83	93.81	93.82	93.81
ERNIE	94.31	94.27	94.27	94.27
ERNIE-CNN	94.18	94.15	94.16	94.15
ERNIE-RNN	93.95	93.94	93.94	93.94
ERNIE-AAFF-SECNN	95.09	95.09	95.08	95.09

Furthermore, the model significantly outperforms approaches using Word2vec embeddings, despite sharing a similar CNN framework. Without pre-training, TextCNN achieves a precision of 90.92% and recall and F1 scores of 90.96%. Notably, ERNIE also surpasses BERT, demonstrating the advantages of domain-adapted pre-training, with improvements of 0.42% in precision, 0.40% in recall and accuracy, and 0.41% in F1 score.

Importantly, although no knowledge distillation or model compression techniques were employed, However, the accuracy of ERNIE-AAFF-SECNN on the 10-class THUCNews dataset is 1.28% higher than that of tinybert, 1.17% higher than that of ALBERT, 1.26% higher than that of MobileBERT, and 1.28% higher than that of DistilBERT. This highlights the effectiveness of the proposed architecture in enhancing the feature representation of short text classification, even when compared with numerous SOTA baselines.

(2) Comparative Results of Classification Experiments on the Today’s Headlines Dataset: On the 13-category Today’s Headlines dataset, hybrid models with different inputs and model structures were used for ensemble training. The comparison models are consistent with those used for the 10-category dataset classification.

As presented in Table 9, the comparative evaluation of various models with different network architectures and input representations is conducted on the Today’s Headlines dataset. The ERNIE-AAFF-SECNN model achieves the highest classification performance, demonstrating substantial effectiveness in handling the 13-category news classification task. It attains precision, recall, F1 score, and accuracy of 91.22%, 91.22%, 91.20%, and 91.22%, respectively—surpassing all baseline models. Compared to the strongest baseline, ERNIE, the proposed model yields improvements of 0.31% in precision, 0.32% in recall and accuracy, and 0.30% in F1 score, underscoring the benefit of its enhanced architecture.

**Table 9:** Experimental results of different models on Today’s headlines.

Model	P %	R %	F1%	Acc %
TextCNN	86.08	86.04	86.02	86.04
TextRNN	85.82	85.68	85.69	85.68
DPCNN	85.68	85.43	85.48	85.43
FastText	87.47	87.32	87.35	87.32
BERT	90.71	90.70	90.79	90.70
BERT-CNN	90.65	90.63	90.63	90.63
TinyBERT	90.69	90.67	90.68	90.67
ALBERT	90.74	90.72	90.73	90.72
MobileBERT	90.70	90.69	90.69	90.69
DistilBERT	90.69	90.68	90.68	90.68
ERNIE	90.91	90.90	90.90	90.90
ERNIE-CNN	90.80	90.77	90.77	90.77
ERNIE-RNN	90.73	90.73	90.70	90.73
ERNIE-AAFF-SECNN	91.22	91.22	91.20	91.22

Conventional models—TextCNN, TextRNN, DPCNN, and FastText—achieve precision values within the range of 85% to 87.5%, with FastText performing best at 87.47%. In contrast, models incorporating pre-trained language representations exhibit a marked performance gain, consistently surpassing 90% across all metrics. Among these, ERNIE achieves the best results with precision of 90.91%, recall and accuracy of 90.90%, and an F1 score of 90.90%. It is worth noting that the proposed ERNEL-AAFF-SECNN also outperforms TinyBERT by 0.55%, ALBERT by 0.5%, MobileBERT by 0.53% and DistilBERT by 0.54% in accuracy, further confirming its superior representation ability.

(3) Bar Chart of Classification Accuracy for Different Models

**Fig 14:**
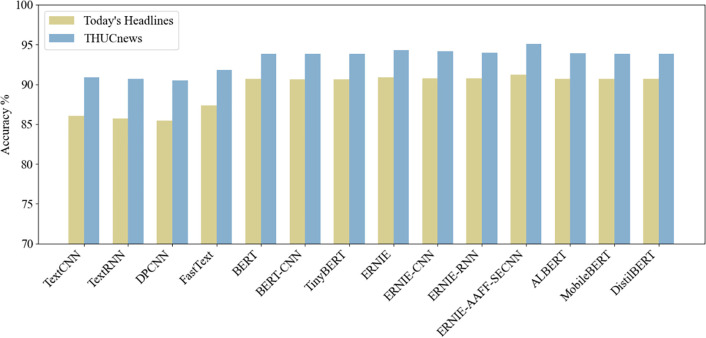
The bar graph accuracy of different models in the THUCNews datasets and Today’s Headlines datasets.

As shown in Fig 14, the bar chart compares the final prediction accuracy of the ERNIE-AAFF-SECNN model with other models on both the THUCNews and Today’s Headlines datasets. It can be observed that the proposed adaptive feature fusion-enhanced model significantly outperforms the comparison models in terms of classification accuracy.

#### 4.4.2 *Comparative results across different categories.*

The classification results for specific categories on the two headline datasets using the proposed large dataset classification model, ERNIE-AAFF-SECNN, are presented in the form of bar charts and metric result tables.

(1) Category results for the THUCNews Chinese headline dataset

Table 10 presents the Classification performance of the proposed model on the 10-category THUCNews dataset, with results averaged over five independent training and evaluation runs. Each metric—Precision, Recall, and F1-score—is presented as mean±standard deviation to reflect performance stability across different random initializations. The model achieves consistently high performance across all categories, with F1-scores above 91% for every class. The Sports category yields the best result (98.70 ± 0.09%), benefiting from distinctive lexical patterns and low semantic ambiguity in sports headlines. Games, Real Estate, and Education also achieve strong F1-scores above 96%, indicating effective feature learning for these topics. Slight performance variation is observed in more semantically ambiguous or overlapping categories. For instance, Stocks achieves an F1 of 91.53 ± 0.22%, which is lower than other financial-related classes like Finance (94.39 ± 0.14%) and Real Estate (95.62 ± 0.15%), likely due to topic mixing in short headlines. Similarly, Technology (91.50 ± 0.18%) shows relatively lower recall, suggesting some misclassification with adjacent domains such as Science or Internet news.

**Table 10:** Classification performance on the THUCNews dataset.

Category	Category label	P %	R %	F1%
Finance	0	93.78 ± 0.23	95.02 ± 0.17	94.39 ± 0.14
Real Estate	1	96.50 ± 0.19	94.82 ± 0.21	95.62 ± 0.15
Stocks	2	93.10 ± 0.27	89.95 ± 0.33	91.53 ± 0.22
Education	3	96.08 ± 0.16	97.15 ± 0.18	96.64 ± 0.12
Technology	4	90.53 ± 0.26	92.45 ± 0.24	91.50 ± 0.18
Society	5	94.84 ± 0.22	94.62 ± 0.20	94.70 ± 0.16
Politics	6	94.42 ± 0.25	92.43 ± 0.26	93.40 ± 0.19
Sports	7	99.09 ± 0.08	98.72 ± 0.10	98.70 ± 0.09
Games	8	96.74 ± 0.17	96.42 ± 0.18	96.60 ± 0.15
Entertainment	9	94.80 ± 0.20	97.95 ± 0.14	96.38 ± 0.16

**Fig 15:**
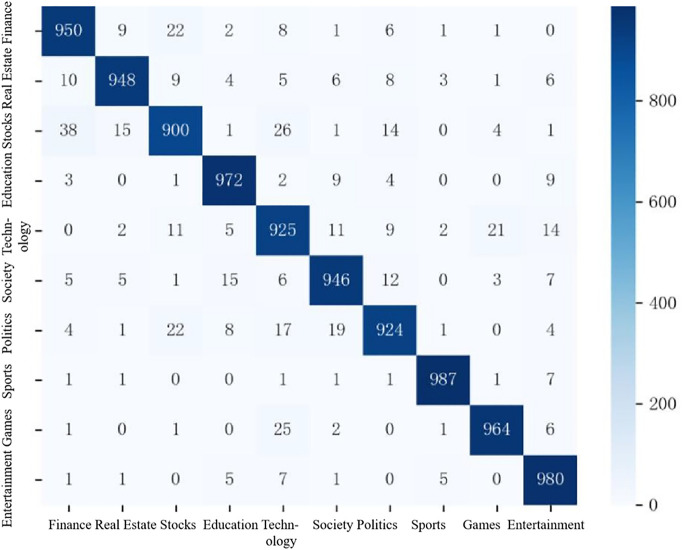
The confusion matrix of ERNIE-AAFF-SECNN on the THUCnews dataset.

To gain deeper insight into classification behavior, a confusion matrix analysis of the model’s predictions on the THUCNews dataset is conducted, as shown in Fig 15.

In the 10-category headline classification task, the model correctly predicts over 900 out of 1,000 samples across all categories, demonstrating consistently high accuracy. The strongest performance is observed in the sports and entertainment domains. For sports, 987 out of 1,000 headlines are correctly classified, with six categories exhibiting only a single misclassification and two categories showing none. In the entertainment category, the model achieves 980 correct predictions, with three categories having no misclassifications and another three containing just one error. These results highlight the model’s exceptional discriminative ability for these topics.

(2) Category results for the today’s headlines dataset

Table 11 presents the per-category performance of our model on the Today’s Headlines dataset, a large-scale benchmark for Chinese short-text classification with 13 fine-grained news categories. Results are averaged over five independent training and evaluation runs, with each metric reported as mean±standard deviation to reflect reproducibility across random initializations. The model achieves strong performance across most categories, with F1-scores exceeding 90% in 8 out of 13 classes. The highest F1 is achieved in Sports (95.62 ± 0.13%) and E-sports (94.71 ± 0.12%), likely due to their distinct vocabulary and low ambiguity in headline-based texts. Real Estate, Military, and Automobiles also show robust results above 93%. Lower performance is observed in Finance (85.11 ± 0.23%) and Technology (87.93 ± 0.25%), indicating challenges in disambiguating subtle economic or technical signals in short, context-limited headlines. International and Tourism exhibit moderate performance, possibly due to semantic overlap with broader categories such as Politics and Society.

**Table 11:** Classification performance on the Today’s headlines dataset.

Category	Category label	P %	R %	F1%
Culture	0	92.73 ± 0.22	90.52 ± 0.24	91.60 ± 0.16
Entertainment	1	91.58 ± 0.27	91.08 ± 0.25	91.33 ± 0.18
Sports	2	94.85 ± 0.19	96.42 ± 0.20	95.62 ± 0.13
Finance	3	85.00 ± 0.33	85.25 ± 0.31	85.11 ± 0.23
Real Estate	4	95.22 ± 0.18	91.28 ± 0.26	93.20 ± 0.17
Automobiles	5	94.16 ± 0.21	93.92 ± 0.22	94.04 ± 0.16
Education	6	89.42 ± 0.26	92.78 ± 0.23	91.07 ± 0.19
Technology	7	89.40 ± 0.32	86.55 ± 0.30	87.93 ± 0.25
Military	8	92.81 ± 0.22	93.88 ± 0.27	93.34 ± 0.18
Tourism	9	87.64 ± 0.28	88.38 ± 0.32	88.00 ± 0.21
International	10	87.29 ± 0.31	88.12 ± 0.26	87.71 ± 0.20
Agriculture	11	89.81 ± 0.24	91.03 ± 0.21	90.41 ± 0.16
E-sports	12	94.66 ± 0.15	95.28 ± 0.20	94.71 ± 0.12

**Fig 16:**
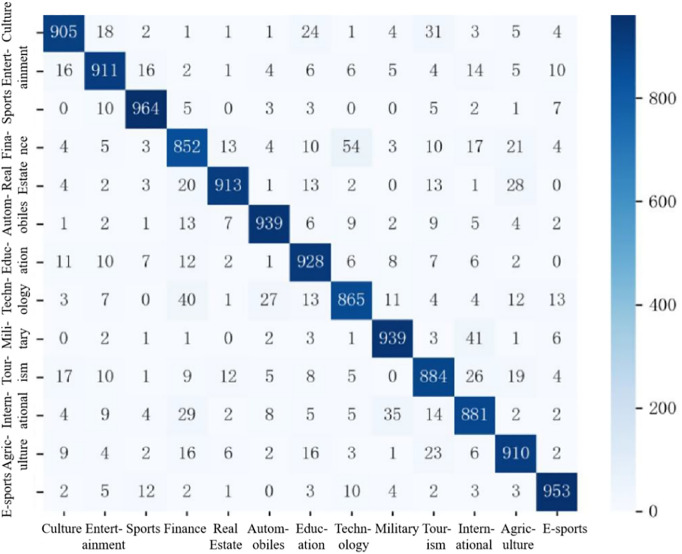
The confusion matrix of ERNIE-AAFF-SECNN on Today’s Headlines datase.

The confusion matrix for the model’s predictions on the Toutiao Chinese headline dataset is presented in Fig 16, providing further insight into its per-class performance and error distribution.

The sports category achieves the highest classification accuracy, with 964 out of 1,000 samples correctly predicted. Misclassifications are minimal, with fewer than 10 instances assigned to any incorrect category, and four categories show no misclassifications at all. The e-sports category also exhibits strong performance, achieving 953 correct predictions, at most 12 misclassified instances across other categories, and perfect classification in one category, demonstrating the model’s high discriminative capability for specialized domains.

#### 4.4.3 *Ablation experiments.*

To validate the effectiveness of each improved module in the ERNIE-AAFF-SECNN model on the two large headline datasets, ablation experiments were conducted using four metrics compared to the unimproved ERNIE-CNN model. The experimental results are shown in Table 12.

**Table 12:** Ablation experiment of ERNIE-AAFF-SECNN.

AAFF	BiLSTM-SE-CNN	THUCnews				Today’s headlines			
		P %	R %	F1%	Acc %	P %	R %	F1%	Acc %
√		94.71	94.70	94.79	94.70	90.85	90.75	90.75	90.75
	√	94.58	94.56	94.56	94.56	91.09	91.08	91.07	91.08
ERNIE-AAFF-SECNN		95.09	95.09	95.08	95.09	91.22	91.22	91.20	91.22
ERNIE-CNN		94.18	94.15	94.16	94.15	90.80	90.77	90.77	90.77

From the table, it can be concluded that compared to the unimproved ERNIE-CNN model, the AAFF module significantly enhances the performance on the 10-category THUCNews headline dataset, with accuracy improving by 0.55%. The BiLSTM-SE-CNN network structure shows a more noticeable improvement in classification performance on the 13-category Today’s Headlines dataset, with accuracy increasing by 0.31%. Removing any module results in a decline in classification performance, demonstrating the effectiveness of the proposed ERNIE-AAFF-SECNN classification model.

### 4.5 *Analysis of experimental results*

#### 4.5.1 *Analysis of comparative experimental results.*

(1) Comparative Results of Classification Experiments on the THUCNews Chinese Headline Dataset

As shown in Table 13, on the THUCNews Chinese headline dataset, hybrid models with different inputs and model structures were used for ensemble training, and the prediction results were evaluated using four metrics: precision (P), recall (R), F1 score (F1), and accuracy (Acc).

**Table 13:** Experimental results of different models in 10% of THUCnews.

Model	P %	R %	F1%	Acc %
TextCNN	86.45	86.05	86.11	86.05
TextRNN	83.34	85.52	82.69	82.52
DPCNN	84.83	84.33	84.31	84.33
FastText	85.84	85.67	85.67	85.67
BERT	91.54	91.46	91.46	91.46
BERT-CNN	91.95	91.90	91.91	91.90
TinyBERT	91.48	91.42	91.45	91.42
ALBERT	92.09	92.03	92.05	92.03
MobileBERT	91.53	91.45	91.48	91.45
DistilBERT	91.50	91.41	91.44	91.41
ERNIE	92.28	92.26	92.26	92.26
ERNIE-CNN	92.15	92.08	92.09	92.08
ERNIE-RNN	92.44	92.38	92.39	92.38
ERNIE-MSSE-DSCNN	94.07	94.04	94.06	94.04

The ERNIE-MSSE-DSCNN model, incorporating adversarial training, achieves a precision of 94.07%, recall of 94.04%, F1 score of 94.06%, and accuracy of 94.04%, outperforming all baseline models. Compared to the best-performing baseline, ERNIE, it demonstrates improvements of 1.92% in precision, 1.78% in recall and accuracy, and 1.95% in F1 score, highlighting the effectiveness of the proposed architecture and adversarial training strategy.

In contrast, the TextCNN model without pre-trained representations achieves lower performance, with precision at 86.45%, recall and accuracy at 86.05%, and F1 score at 86.11%. This significant performance gap underscores the critical role of pre-trained language models in enhancing text classification accuracy. Moreover, ERNIE surpasses BERT-CNN by 0.33% in precision, 0.36% in recall and accuracy, and 0.35% in F1 score, further validating the advantage of domain-optimized pre-training for Chinese text understanding.

It is worth noting that in the low-resource setting with only 10% training data, ERNIE-MSSE-DSCNN outperforms the compact SOTA model TinyBERT by 2.62% and ALBERT by 2.01% in accuracy. It outperforms MobileBERT by 2.59% and DistilBERT by 2.63%, demonstrating outstanding robustness and learning efficiency even with extremely limited labeled data.

(2) Comparative results of classification experiments on the today’s headlines dataset

As summarized in Table 14, the performance of various models with different architectures and input representations is evaluated on the 13-category Today’s Headlines dataset. The proposed ERNIE-MSSE-DSCNN model, enhanced with adversarial training, achieves the highest classification performance, with precision, recall, F1 score, and accuracy of 88.71%, 88.68%, 88.69%, and 88.68%, respectively. It has an accuracy rate 0.83% higher, a recall rate 0.83% higher, a precision 0.84% higher, and an F1 score 0.86% higher than that of the best baseline ERNIE-CNN. The scores of the ERNIE-CNN baseline on the four indicators were 87.87%, 87.85%, 87.83% and 87.85% respectively. Although the integration of ERNIE embedding with the standard CNN structure provided measurable performance gains on this small-scale multi-class dataset, the results were still inferior to those of the proposed model. This confirms that the improvements in network design – especially the MSSE and DSCNN components – have significantly enhanced the accuracy of feature representation and classification.

**Table 14:** Experimental results of different models on 10% of Today’s headlines.

Model	P %	R %	F1%	Acc %
TextCNN	81.32	80.98	81.02	80.98
TextRNN	76.00	73.69	74.05	73.79
DPCNN	77.44	76.71	76.88	76.71
FastText	78.75	78.60	78.62	78.60
BERT	85.82	85.69	85.69	85.69
BERT-CNN	86.51	86.51	86.44	86.43
TinyBERT	85.23	84.92	85.08	84.92
ALBERT	86.26	86.11	86.19	86.11
MobileBERT	85.74	85.69	85.72	85.69
DistilBERT	85.47	85.43	85.44	85.43
ERNIE	87.38	87.16	87.16	87.16
ERNIE-CNN	87.87	87.85	87.83	87.85
ERNIE-RNN	87.57	87.55	87.54	87.55
ERNIE-MSSE-DSCNN	88.71	88.68	88.69	88.68

It is worth noting that with a low resource setting of only 10% of the Today ‘s Headlines dataset, the accuracy of ERNIE-MSSE-DSCNN is 3.76% higher than that of TinyBERT and 2.57% higher than that of ALBERT. It outperforms MobileBERT by 2.99% and DistilBERT by 3.25%, highlighting its outstanding generalization ability when labeled data is severely limited.

(3) Bar chart of classification accuracy for different models

**Fig 17:**
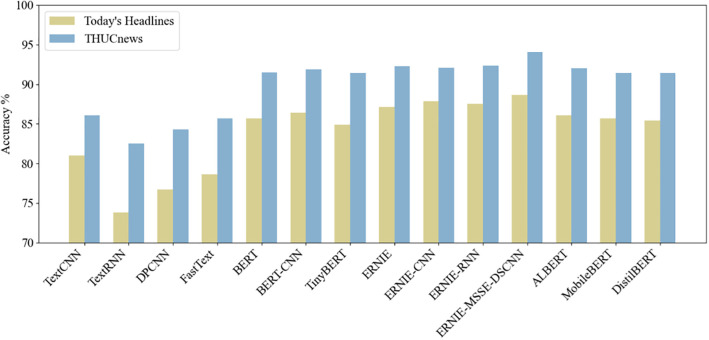
The bar graph accuracy of different models in the THUCNews datasets and Today’s Headlines datasets.

As shown in Fig 17, the bar chart compares the final prediction accuracy of the proposed model with other models on both the THUCNews and Today’s Headlines datasets. From the chart, it can be observed that the ERNIE-MSSE-DSCNN classification model, constructed using the FGM strategy for adversarial training, achieves higher accuracy on datasets with different numbers of categories compared to other models.

#### 4.5.2 *Comparative results across different categories.*

The classification results for specific categories on the two headline datasets using the proposed large dataset classification model, ERNIE-MSSE-DSCNN, are presented in the form of bar charts and metric result tables.

(1) Category results for the THUCNews dataset

Table 15 presents the per-class performance of our model on the standard 10-category THUCNews dataset, with results averaged over five independent training and evaluation runs. Each metric—Precision, Recall, and F1-score—is reported as mean±standard deviation to reflect consistency across different random seeds. The model achieves strong performance across all categories, with F1-scores exceeding 92% in 8 out of 10 classes. The highest F1 is observed in Sports (98.51 ± 0.10%), followed by Education (96.70 ± 0.14%) and Entertainment (95.81 ± 0.17%), indicating excellent discrimination for topics with distinctive lexical patterns. Real Estate and Games also perform robustly above 95%. Lower performance is seen in Stocks (88.60 ± 0.22%) and Technology (91.88 ± 0.19%), suggesting challenges in disambiguating fine-grained financial or technical content from short headlines. The relatively lower precision in Finance (94.30 ± 0.23%) may stem from semantic overlap with Stocks and broader economic news.

**Table 15:** Classification performance on 10% of the THUCnews dataset.

Category	Category Label	P %	R %	F1%
Finance	0	94.30 ± 0.23	91.20 ± 0.25	92.72 ± 0.16
Real Estate	1	96.55 ± 0.18	95.05 ± 0.24	95.78 ± 0.15
Stocks	2	87.02 ± 0.31	90.25 ± 0.33	88.60 ± 0.22
Education	3	96.42 ± 0.17	96.98 ± 0.21	96.70 ± 0.14
Technology	4	89.70 ± 0.29	91.05 ± 0.26	91.88 ± 0.19
Society	5	93.48 ± 0.20	94.25 ± 0.22	93.86 ± 0.15
Politics	6	93.50 ± 0.26	91.12 ± 0.28	92.29 ± 0.18
Sports	7	98.98 ± 0.09	98.05 ± 0.12	98.51 ± 0.10
Games	8	96.80 ± 0.19	95.04 ± 0.20	95.90 ± 0.16
Entertainment	9	94.20 ± 0.24	97.48 ± 0.18	95.81 ± 0.17

**Fig 18:**
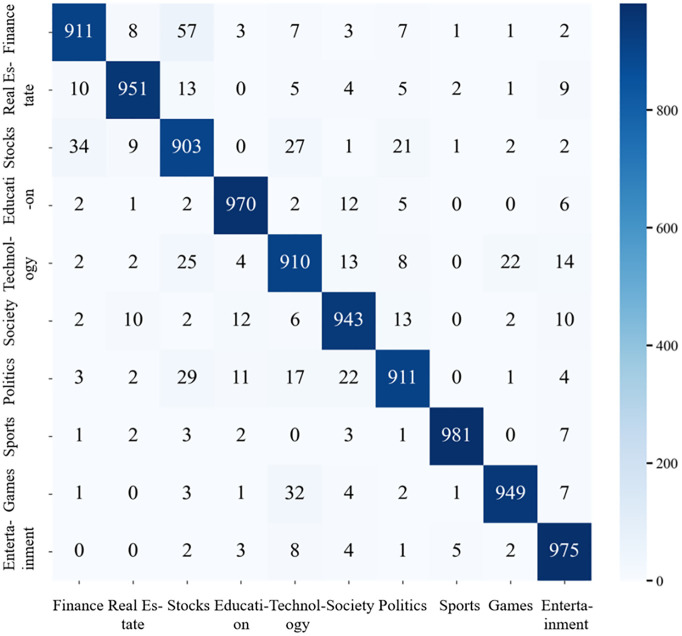
The confusion matrix of ERNIE-MSSE-DSCNN on 10% of the THUCnews dataset.

To gain deeper insights into the model’s performance on the THUCNews dataset, the prediction results are analyzed via the confusion matrix shown in Fig 18. The sports category achieves the highest accuracy, with 981 out of 1,000 headlines correctly classified and fewer than 7 misclassifications across all other categories. The entertainment category also exhibits strong performance. Notably, for all 10 categories, the number of accurately predicted samples exceeds 900, demonstrating the model’s consistent and robust classification capability across diverse topics.

(2) Category results for the today’s headlines dataset

Table 16 reports the class-wise performance of our model on the Today’s Headlines dataset, a large-scale benchmark for fine-grained Chinese short-text classification with 13 categories. Results are averaged over five independent runs, and each metric is presented as mean±standard deviation to reflect reproducibility across different random seeds. The model achieves strong performance in several domains, with F1-scores exceeding 90% in 7 out of 13 classes. The highest F1 is observed in Sports (94.37 ± 0.15%) and E-sports (93.55 ± 0.13%), likely due to their distinct vocabulary and clear topic boundaries in headline texts. Real Estate, Automobiles, and Military also perform robustly above 90%. Lower performance is seen in Finance (83.01 ± 0.24%) and Technology (84.72 ± 0.25%), indicating challenges in disambiguating subtle economic or technical signals from short, context-limited headlines. Tourism (83.44 ± 0.23%) and International (84.56 ± 0.21%) exhibit moderate results, possibly due to semantic overlap with broader categories such as Society and Politics. The small standard deviations (ranging from 0.13 to 0.34 percentage points) demonstrate high consistency across runs, confirming the model’s stability. The macro-averaged F1-score across all categories is 88.85 ± 0.21%, validating the effectiveness of our approach on this challenging multi-class news classification task.

**Table 16:** Classification performance on 10% of the Today’s headlines dataset.

Category	Category Label	P %	R %	F1%
Culture	0	87.45 ± 0.26	88.82 ± 0.24	88.13 ± 0.17
Entertainment	1	90.08 ± 0.28	88.78 ± 0.27	89.42 ± 0.19
Sports	2	93.20 ± 0.20	95.58 ± 0.23	94.37 ± 0.15
Finance	3	80.22 ± 0.34	85.98 ± 0.32	83.01 ± 0.24
Real Estate	4	93.40 ± 0.21	89.75 ± 0.25	91.52 ± 0.18
Automobiles	5	94.75 ± 0.19	92.52 ± 0.22	94.12 ± 0.16
Education	6	89.92 ± 0.27	88.98 ± 0.26	89.44 ± 0.18
Technology	7	85.95 ± 0.33	83.55 ± 0.31	84.72 ± 0.25
Military	8	90.13 ± 0.24	90.38 ± 0.26	90.25 ± 0.19
Tourism	9	86.40 ± 0.30	80.68 ± 0.33	83.44 ± 0.23
International	10	83.00 ± 0.32	86.22 ± 0.28	84.56 ± 0.21
Agriculture	11	89.60 ± 0.25	88.22 ± 0.23	88.90 ± 0.17
E-sports	12	93.43 ± 0.16	93.68 ± 0.19	93.55 ± 0.13

**Fig 19:**
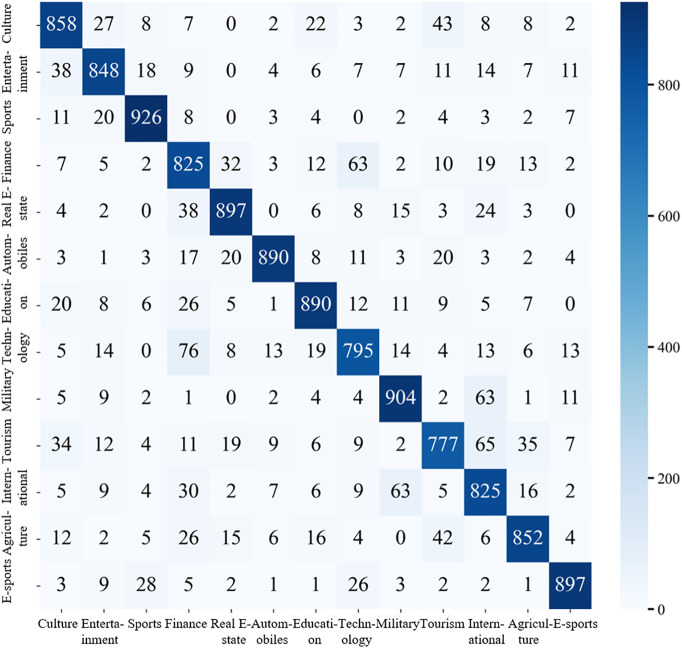
The confusion matrix of ERNIE-MSSE-DSCNN on 10% of the Today’s Headlines dataset.

The confusion matrix of the prediction results for the 10%-scale dataset using the ERNIE-MSSE-DSCNN model is analyzed, as shown in Fig 19.

This model demonstrates excellent classification performance in the fields of sports and e-sports. In sports, 926 out of 1,000 samples were correctly classified, with the fewest misclassifications – only two categories had no mispredictions. In addition to the tourism industry with 777 correct predictions and the Technology industry with 795 correct predictions. All the other 11 categories achieved at least 825 correct classifications. These results highlight the model’s strong generalization ability on various topics, especially in professional fields such as sports and Military.

#### 4.5.3 *Ablation experiments.*

To validate the improvements of the proposed ERNIE-MSSE-DSCNN model, ablation experiments were conducted using four metrics compared to the unimproved ERNIE-AAFF-SECNN model. The experimental results are shown in Table 17.

**Table 17:** Ablation experiment of ERNIE-MSSE-DSCNN.

MSSE-DSCNN	FGM Adversarial Training	THUCnews				Toutiao			
		P %	R %	F1%	Acc %	P %	R %	F1%	Acc%
√		93.13	93.11	93.11	93.11	88.28	88.12	88.13	88.12
	√	93.35	93.28	93.30	93.28	88.33	88.32	88.32	88.32
ERNIE-MSSE-DSCNN		94.07	94.04	94.06	94.04	88.71	88.68	88.69	88.68
ERNIE-AAFF-SECNN		91.75	91.72	91.66	91.72	87.18	87.14	87.14	87.14

The results indicate that, for both small datasets, the proposed enhancements—specifically the MSSE-DSCNN module and FGM-based adversarial training—effectively improve classification performance. Incorporating the MSSE-DSCNN module boosts accuracy by 1.39% and 0.98% on the respective datasets. The integration of FGM adversarial training yields an even more pronounced improvement, increasing accuracy by 1.56% and 1.18%, respectively.

## 5 Conclusion

The value of classifying Chinese news headlines lies in its ability to efficiently identify and categorize a large volume of news information, helping users quickly locate content that aligns with their personal interests, thereby optimizing information retrieval and reading experiences. Building on the discussion of the research background and significance, we analyzed relevant techniques and algorithms for short text classification. To address the challenges faced in classifying Chinese news headline datasets, such as feature sparsity, domain-specific terminology, significant length variations, and information loss due to tokenization or truncation, we constructed two tailored models: the ERNIE-AAFF-SECNN for large datasets and the ERNIE-MSSE-DSCNN for small-scale datasets. In the following, we validate these advancements through empirical analysis:

Now, based on the empirical results, we have summarized and verified our four main contributions:

1. Scale adaptive data preprocessing: To address the challenge of limited data in small-scale datasets, we have applied an improved enhanced strategy based on AEDA. As shown in Table 3, the enhanced samples retain the original label semantics. This qualitative improvement helps to alleviate overfitting during training, especially when labeled data is scarce. For large-scale datasets, length filtering ensures computational efficiency, reduces redundant information in long titles, and contributes to more stable model learning.
2. Attention-based Adaptive Feature Fusion (AAFF): The ablation study (Table 12) indicates that on the THUCnews dataset, removing AAFF leads to a reduction of 0.63%, 0.53%, 0.55%, and 0.55% in F1, P, R, and Acc, respectively. This confirms the crucial role of AAFF in capturing meaningful hierarchical character semantics from ERNIE output.
3. Adversarial training with FGM: The ablation experiment (Table 17) indicates that when applied to small datasets, FGM enhances the model’s robustness and reduces overfitting. On the THUCnews dataset, F1, P, R and Acc increased by 1.64%, 1.6%, 1.56% and 1.56% respectively. On the Toutiao dataset, F1, P, R and Acc increased by 1.18%, 1.15%, 1.18% and 1.18% respectively.
4. Enhanced classification architecture: Our improved TextCNN features the MSSE mechanism and depthwise separable convolution, achieving outstanding performance. The comparative experiment (Table 14) shows that compared with the standard TextCNN, F1, P, R and Acc have increased by 7.67%, 7.39%, 7.7% and 7.7% respectively.

## 6 Limitations and future work

Despite the promising results, this study has several limitations that suggest directions for future research. First, the current framework focuses only on news headlines, which are often too short to fully capture domain-specific semantics. This limits the model’s ability to disambiguate terms that appear across multiple categories. Future work should explore integrating full-text content to enhance contextual understanding and improve cross-domain classification performance.

Second, the proposed models are tailored for different data scales—small-scale and large-scale datasets—requiring manual selection based on prior knowledge of data volume. To improve generalization, we plan to develop an automatically adaptive classification system that dynamically chooses optimal feature extraction methods and model architectures according to the input data size. Such a system would enhance flexibility and applicability in real-world scenarios.

In summary, by advancing domain-aware learning and building adaptive frameworks, we aim to further improve the accuracy and efficiency of short news title classification for practical deployment.

## Supporting information

S1 Data Data. (ZIP)
